# Research and practice of the BOPPPS teaching model based on the OBE concept in clinical basic laboratory experiment teaching

**DOI:** 10.1186/s12909-023-04822-z

**Published:** 2023-11-17

**Authors:** Pei Li, Xiaopeng Lan, Lin Ren, Xiaoping Xie, Haitao Xie, Shuangquan Liu

**Affiliations:** Department of Clinical Laboratory Medicine, Medical Laboratory Teaching Center, The First Affiliated Hospital, Hengyang Medical School, University of South China, No. 69, Chuanshan Road, Hengyang City, 421001 Hunan China

**Keywords:** Clinical basic laboratory, OBE, BOPPPS, Teaching model, Experimental teaching

## Abstract

"Clinical basic inspection technology" is one of the essential courses in the medical laboratory profession. Combining the characteristics of the discipline itself, the research and practice of the BOPPPS model based on the OBE concept in clinical basic laboratory experiment teaching are discussed, and the reform of in teaching objectives, teaching contents, and teaching design path is implemented. The "student-centered" teaching process is divided into six stages: before, during, and after class, and the teaching process is continuously improved to achieve the desired teaching effect. Results of the experiment teaching show that the model has improved students' active participation and developed their clinical thinking skills, and more than 95% of students are satisfied with this teaching model.

## Background

Medical laboratory science is an important branch of clinical medicine and a comprehensive discipline that intersects, penetrates, and integrates basic and clinical medicine. The course titled "Clinical basic laboratory" is one of the important courses for medical laboratory majors, with many knowledge points, wide coverage, complex morphology content, and strong professional relevance. It requires students to pay extra attention to the linking of theoretical knowledge and practical operation and to have strong analytical and problem-solving skills [1]. The main teaching content of this course involves the physical and chemical examination of blood, urine, feces, and other body fluids as well as the physiology and morphology of various humoral cells and tissues. Morphology is not only an important item of clinical examination but also the "gold standard" for the diagnosis of some diseases [2].

Currently, curriculum teaching entails the following difficulties: 1) the teaching method is a single, traditional classroom, whereby the teacher has an excessive teaching load, students fail to memorize and understand abstract theoretical knowledge, and their learning interest is not high enough; 2) experimental classroom teaching mainly involves validation experiments, as complicated tests and experimental designs are few, whereby students lack research consciousness and innovation spirit [3]; 3) since cell morphology is difficult to grasp and the morphology and internal structure of the cell are complex and variable, these lead to boredom and tediousness in students’ learning; and 4) single teaching evaluation fails to assess the comprehensive ability of students. In view of these problems in teaching, exploring new teaching models is both very important and urgent.

To resolve this teaching dilemma, after many discussions, the research group decided to introduce the OBE (outcome-based education) concept BOPPPS (bridge-in, learning objective, pretest, participatory learning, posttest and summary) in the teaching model of basic clinical laboratory medicine. OBE was created by SPady in 1981 and is highly sought by academic circles worldwide [4]. OBE requires the core concepts student-centered, outcome-oriented, and continuous improvement. The whole teaching implementation process is based on the students' learning results, the starting point for designing the professional training objectives. All teaching design, teaching content, and teaching implementation must focus on these teaching objectives. Students' learning outcomes are obtained through professional teaching activities, extracurricular activities, academic activities, and so on. These include not only professional knowledge, practical operation, and comprehensive ability, but also an outlook on life, values, professional identity, and sense of mission. The OBE concept has been applied in talent training, professional development, and curriculum construction in China's higher education institutions [5–8], while all existing theoretical studies show that OBE is an inevitable choice to improve the quality of talent training.

The BOPPPS teaching model was developed by Douglas Kerr's team in Canada and has since been replicated in more than 30 Western countries [9, 10]. BOPPPS emphasizes that different teaching objects adopt different methods and means to achieve teaching objectives and emphasizes the initiative of students in learning, the interaction between teachers and students, and the integrity of teaching links. To achieve learning objectives, the teaching implementation process is divided into six segments (Table 1): bridge-in (B), objective (O), preassessment (P), participatory learning (P), postassessment (P) and summary (S). These six elements of this teaching model are interlocked and closely linked to teaching activities. Teachers can refine and decompress teaching content into the teaching process. Through participatory learning, students can flexibly use their knowledge to analyze and solve problems, improving learning interest, exploring the blind spots of learning, and ultimately improving the learning effect [11]. BOPPPS provides an effective method for classroom teaching reform.

**Table 1:** Boppps teaching model

Implementation link	Mode	Purpose
Bridge-In	Introduced with hot events, news, and classic cases	Improve students' interest in learning
Objective	Teachers's rspective: course objectives, key and difficult points Student rspective: learning objectives; students can self-evaluate whether they meet the requirements after learning	Teacher's view: clear learning objectives, to facilitate students to master the learning focus Students' perspective: guide the direction of students' learning, tell students what I need to learn and how to learn
Pre-Test	Questionnaire, ask questions, or test question form	Master each students' autonomous learning ability,
Participatory Learning	Group discussion, role playing, case analysis, seminars, etc	Important Means of Cultivating Students' Active Participation in Learning
Post-Test	Small test, comprehensive experimental operation, comprehensive case analysis, discussion, PPT display, etc	Teachers' perspective: Evaluate the teaching process, reflect on teaching, and adjust Student perspective: keep abreast of their mastery of knowledge
Summary	A lesson mind map, summary	Teachers’ guide; students summarize their knowledge

The guiding practice of the learning outcome-oriented OBE concept has disrupted the traditional education concept. It is a reverse design and positive implementation based on the acquisition of students' expected ability and focuses on “student output.” The BOPPPS teaching model focuses on what students have “learned” rather than what teachers have “taught” them and fully realizes the student-centered teaching method, in line with the OBE concept. The introduction, objective, and pretest of BOPPPS reflect what to learn and why to learn in the OBE concept. Participatory learning reflects how to learn in the OBE concept, the post-test, and summary and how to echo the learning in the OBE concept. The teaching model of BOPPPS based on the OBE concept is thus introduced to reform the traditional teaching model, improve students' ability, guide students to develop advanced knowledge exploration, and cultivate students' awareness of independent learning.

In recent years, domestic university disciplines have combined the OBE concept with the BOPPPS teaching model, e.g., orthodontics, pathogenic biology and immunology, thoracic surgery and medical information literacy courses [12–15]. However, there is still no report on the application of the OBE concept and the BOPPPS teaching model in the “Clinical basic laboratory” course. Although the OBE concept and the BOPPPS teaching model have proven successful and improve students' comprehensive ability, it remains unclear whether they can be effective for medical laboratory science students to achieve “student-centered” teaching purposes.

## Methods

### Study subjects

The laboratory medicine major is a clinical medical technology major, and only one class is enrolled in this topic each academic year. This study included 92 medical laboratory undergraduates across two academic years including 47 students from the 2017 class and 45 students from the 2018 class.

The students of laboratory medicine in these two years came from all regions of China, and more than half of them were native to the Hunan Province (63.8% and 60% in 2017 and 2018, respectively). There were 27 girls and 20 boys in the 2017 class, with an average age of 21.6. There were 24 girls and 21 boys in the 2018 class, with an average age of 21.3. There were no significant differences in gender, age, family background, or entrance achievement between the two groups (*P* > 0.05). They all received systematic precollege education under the same guidelines and used the same textbook while passing the entrance examination requirements. The lecturers were all from the Laboratory Medicine Center of the First Affiliated Hospital of the University of South China, and the teaching chapters of the 4 teachers were also the same.

In the traditional teaching method in grade 2017, the teacher taught in the classroom, and the students passively listened to these lectures and carried out experimental operations step by step. Finally, the teacher summarized the class, and the students completed their homework after class.

The students in grade 2018 adopted the BOPPPS online and offline blended classroom teaching model. This course lasted 96 class hours over 12 weeks with 8 lessons per week and 45 min per class. The experiments in this course required the use of a microscope to observe the morphology of different specimens and cells. The chapter on blood smear staining, for example, it involved 12 h.

All the teachers were from the Laboratory Medicine Center of the First Affiliated Hospital of University of South China. The teaching content of the chapters used by the teachers in the two academic years was roughly the same. All the teachers had at least 5 years of teaching experience and met the standard requirements for teaching in higher education institutions.

#### OBE concept and BOPPPS teaching model in reform idea teaching

### Teaching objectives

These objectives aim to establish the training objectives and graduation requirements for clinical basic laboratory science according to the job requirements of hospitals and third-party laboratories. The OBE focuses on student outcomes in the teaching process. Therefore, before teaching, teachers need to clarify why the students are taking the course, what they can learn, and what competencies they can acquire. The overarching objective of the "Clinical" course is to eventually become technical and practical personnel by establishing the following practical teaching objectives: to enable students to consolidate the basic principles, concepts, and related knowledge learned in the theoretical part of the course and to master the basic operation and use skills of basic clinical laboratory techniques. Students thus develop professional ethics, establish a sense of professional identity and responsibility, comply with laboratory procedures and biosafety, and acquire the ability to analyze and interpret experimental results, laying a solid foundation for clinical practice.

#### Knowledge objectives

These objectives target the mastery of the methods, principles, quality control, and methodology evaluation of routine items. They also aim to promote familiarity with the three routine inspections of professional standards, technical standards, and relevant laws and regulations.

#### Competency objectives

These objectives include the acquisition of blood specimen collection and anticoagulation techniques, blood smear preparation and staining techniques, and blood cell counting techniques through participatory learning. Others include mastery of the techniques of the morphological identification of cells, eggs, crystals, and tubular forms in clinical specimens; mastery of the use of the microscope, blood cell analyzer, urine analyzer, stool analysis workstation, and other instruments and equipment; and the ability to think clinically about, analyze, and solve practical clinical problems.

#### Quality objectives

These objectives include establishing a correct outlook on life and values with a practical, rigorous, and down-to-earth style of work and a professional spirit of fear for life and love for work; becoming patient-centered; improving the quality and level of medical services; obtaining a good sense of teamwork and the ability to communicate and express themselves to doctors and patients; and encouragement to participate in teaching competitions and academic reading to develop scientific literacy and innovation.

### Teaching content

The teaching content is arranged around the teaching objectives according to the epistemology of "theory–practice-retheory-practice." It aims to teach how to effectively learn knowledge and constantly deepen students' overall grasp of the basic theoretical system of clinical examination. The basic practical teaching content is integrated, and open, interesting, and innovative experiments are added. For example, in the experimental course, negative or positive specimens are randomly distributed, allowing students to design their experiments and publish their experimental results. According to the teaching characteristics of clinical examination morphology, teachers add a small program of self-testing for map recognition, maximize the advantages of a digital microscope, and launch interactive morphology discussions, quiz competitions, group teaching, and examinations with students. We also add clinical cases and report discussions to combine theory and clinical practice, e.g., concerning the cause of thrombocytopenia or the "uninvited guest" in urine.

### Teaching design and practice: the exemplar section on "blood smear staining and microscopic examination"

**Fig. 1:**
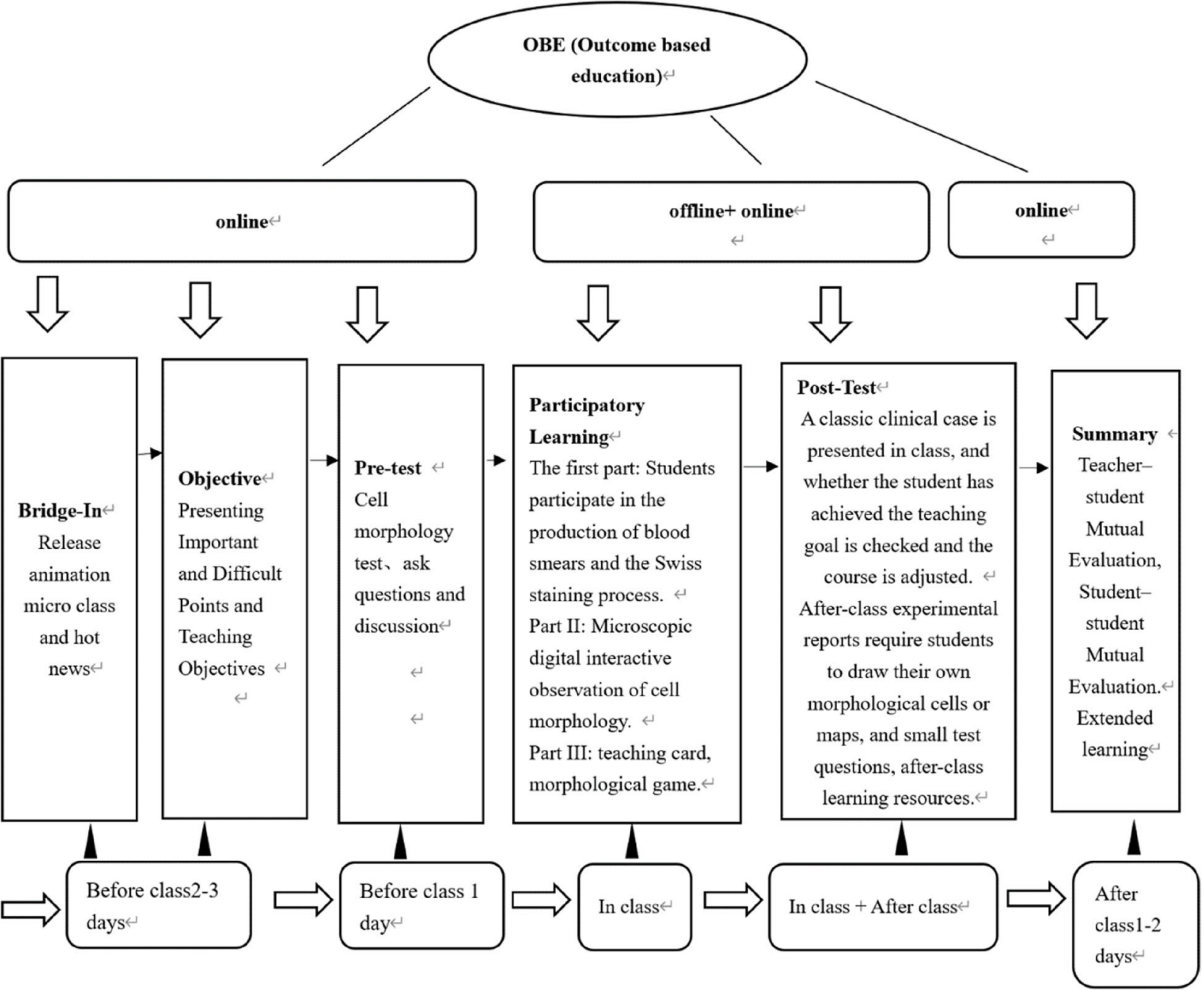
Boppps teaching design: the section on "blood smear staining and microscopic examination" as an example

Guided by postcompetency, a teacher completed the three teaching stages before, during, and after this class through the teaching design of six links of BOPPPS. According to the teaching characteristics of this class, the teaching design of the six links of BOPPPS was slightly adjusted. Through hands-on operation and digital microscope observation, we can deepen the understanding of peripheral blood cell morphology (Fig. 1). The knowledge objectives in this section are mastery of the basic concepts of peripheral blood cell testing, of the basic principles of methodology, of the preparation and staining methods of blood smears, and of laboratory quality assurance. The ability objectives include preparation and staining of blood smears, comprehensive experiment design, peripheral blood cell morphology recognition and clinical application ability.

#### Bridge-in

To help students complete the preclass preview in the learning app, better understand the new knowledge points, and improve their interest and enthusiasm toward classroom learning, a teacher posted an animated microlesson on the production of blood smears, and the principles of Wright stain were introduced in class. Additionally, recent viral news stories related to this course, such as problems with blood cell morphology testing and clinical experiments, were presented in class to motivate students to feel a sense of mission and responsibility for their future careers.

#### Objective

The key points, difficult knowledge, and teaching objectives of the lesson are released in e-learning software in the form of a PPT to grant students a guiding direction to understand what they should learn and why they should learn it.

#### Pre-test

**Fig. 2:**
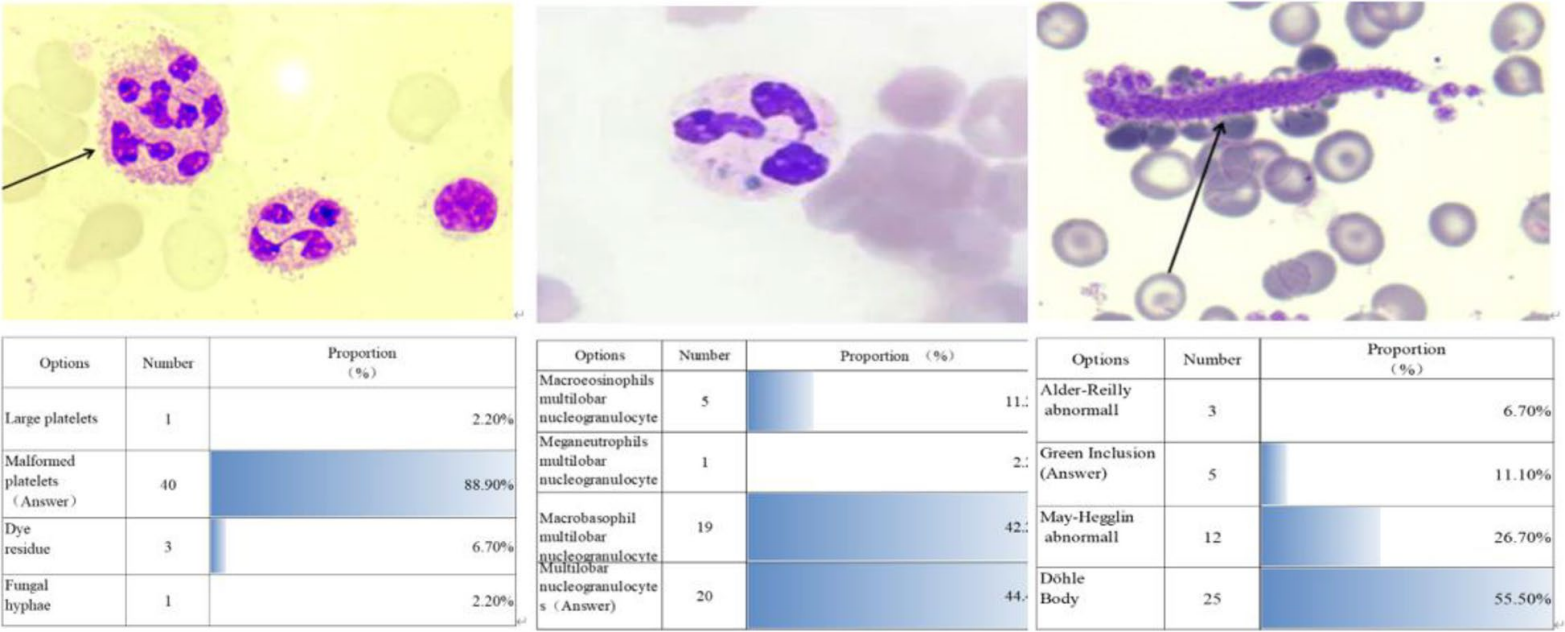
System of the literacy self-assessment app

Some concepts of normal cell morphology were mastered in the previous period. This classroom used flash technology; teachers published self-test questions for learning app recognition, and students tested themselves. According to the scoring of the backstage system of the literacy self-assessment app, teachers can better grasp their students' prior learning (Fig. 2). Teachers post these questions online to grasp whether their students' prestudy has been completed. According to the pretest situation, teachers obtain students' feedback for the first time and make adjustments to the teaching content and the design of important and difficult points for learning analysis.

#### Participatory learning

Teacher-led, student-centered learning was divided into three parts. In the first part, the teachers randomly distributed blood samples from students' own blood smear operation and Wright staining. According to the five steps of blood smear, one take, two dips, three oblique, four pull, and five push, the teacher explained the precautions. Students supervised each other concerning the pushing effect in terms of whether pushing thick, short, body, and tail junction was clear and that showing “snake” did not meet the requirements of the repush. In the second part, the teacher explained the principles and precautions of Wright's staining. The students dyed themselves and dried the specimens. The cell morphology was observed through a digital microscope. Given that their specimens were randomly distributed, the students observed and compared each other’s samples. Teachers took photos through a digital microscope and guided students to observe and address the competition in groups. The third part involved deepening abstract morphological understanding. Teachers prepared teaching pictures and had the students participate in a game of morphological breakthroughs. Dragging the correct picture to the correct answer could be the next breakthrough. This game was designed with levels from easy to difficult. Students participated in the whole process of interaction to deepen their understanding, thereby improving their learning interest in morphology.

#### Post-test

This test is divided into two parts: in class and after class. A classic clinical illness is presented in a post-test class, e.g., to identify a case of vomiting caused by thinking. Through comprehensive case analysis, students develop the ability to analyze problems, solve problems, and think clinically. Teachers can grasp the whole teaching effect of students through their answers and feedback and based on whether they have reached the teaching objective of cost-saving classes. The after-class post-test adopts the form of homework and using the learning app for practice questions. This homework requires students to draw or paste pictures themselves to deepen their study of morphology, rendering boring homework interesting. After class, teachers share learning materials with students to expand and deepen their knowledge.

#### Summary

This process includes teacher summary and student summary. Teacher summary knowledge points, through charts, indicate the shortcomings of the teaching process and offer teaching reflection. Teachers must answer students' questions, and the whole class teaching design may be subject to flexible adjustment, constantly optimizing the teaching plan and continuously improving to add vitality to the classroom and increase students' learning confidence. Student summary allows students to build their mind maps in reviewing the key and difficult knowledge in this class, consolidating the knowledge of the whole class while refining the content. According to the evaluation criteria of the teaching objectives, they self-evaluate and summarize their learning status.

### Evaluation

The teaching evaluation of results-oriented OBE mainly focuses on student learning outcomes, which need to be evaluated through how students "learn". To better evaluate the feasibility of the BOPPS model, the assessment of the 2017 students and the 2018 students was compared after their courses as follows: 100 points for the laboratory exam, including the operation test and the blood cell morphology test; 100 points for the written test (the content includes knowledge points of blood tests);, and randomly selected questions from the question bank including single-choice questions, multiple-choice questions, short answer questions (5 points per small question, 5 small questions, a total of 25 points) and comprehensive case analysis questions (10 points per small question, 2 small questions, a total of 20 points). Finally, the overall score was integrated into a 100-point scale (30% for laboratory tests and 70% for written tests).

In addition, before the exam, online grading for the 2018 class was collected before, during and after class to assess students' daily learning. The scores included answers to questions on the learning app (30 points), discussion and questions (10 points), homework (30 points), attendance class (10 points), and watching learning videos (20 points).

Finally, a questionnaire survey was conducted in the 2018 class to analyze the impact of BOPPPS on students' learning abilities, e.g., finding problems, analyzing problems, solving problems, or teamwork. A total of 45 valid questionnaires were collected and analyzed (Table 2). Through the quantitative evaluation of course objectives, the evaluation of course "teaching" was analyzed, and students' evaluations of their achievements of learning goal expectations were examined.

**Table 2:** Questionnaire table

Survey items	Very Willing(%)	Willing(%)	Unwilling(%)	Very Unwilling(%)
Satisfaction with the teaching model	66.60	31.10	2.30	0.00
Memorization and understanding	51.10	42.20	4.40	2.30
Interaction between teachers and students	57.70	40.00	2.30	0.00
Breadth and depth of knowledge acquisition	62.20	31.10	4.40	2.30
Creative spirit and awareness of research	60.00	28.80	6.60	4.60
Learning is more planned and effective	55.50	31.10	8.80	4.60
Clinical thinking skills improved	60.00	28.80	6.60	4.60
Self-directed learning ability	53.30	40.00	4.40	2.30
Effective guidance and management of teachers	57.70	31.10	8.80	2.40
Increase learning interest	62.20	28.80	6.60	2.40
improve clinical communication skills	60.00	35.50	4.50	0.00
Digital microscope experience	62.20	33.30	4.50	0.00
Summary, feedback	57.70	35.50	4.40	2.40

### Data analysis

All analyses were performed using GraphPad Prism 8.0. Statistical Analysis. Data were expressed as the mean ± standard deviation, and statistical significance was determined using an unpaired Student t test for the two groups and ANOVA for multiple groups. Pearson correlation was performed between individuals’ final score and the online score. The degree of objective attainment data analysis is a correlation coefficient test. Statistical significance was set at *P* < 0.05.

## Results

### Students’ examination scores

**Fig. 3:**
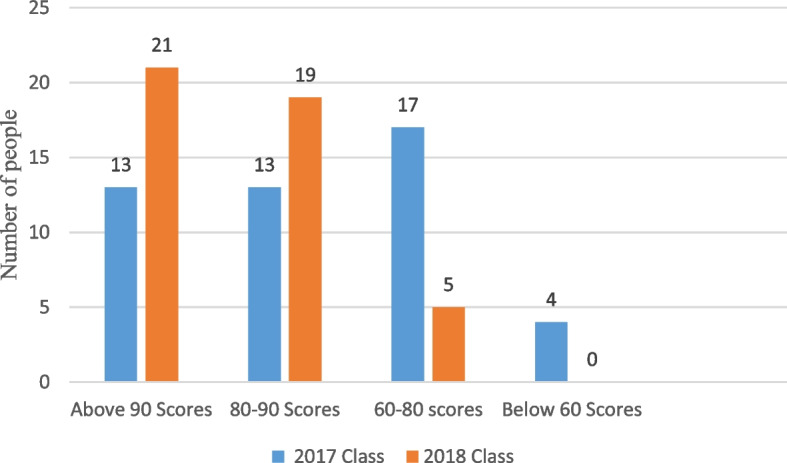
BOPPPS teaching model based on OBE concept Column distribution of an experimental 2017 class and a traditional 2018 class

The author applied the OBE concept and BOPPPS teaching model to the final examination results of the 2018 medical laboratory class blood knowledge test, which were significantly higher than 47 students in the 2017 medical laboratory traditional class. The 2018 class comprised 45 students. A total of 21 people had more than 90 points in their examination results, 19 people had 80–90 points, 5 people had 60–80 points, and 0 people failed (Fig. 3).

**Fig. 4:**
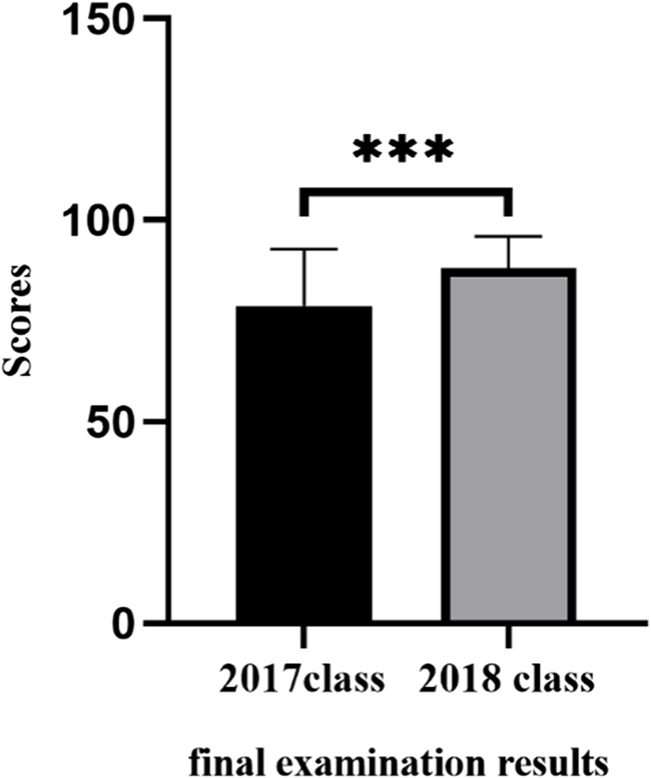
The grade identification of the fourth diagram is the opposite. The bar chart on the left should show the 2018 grade, and the bar chart on the right shows the 2017 grade

The final examination scores of the students enrolled in the 2018 class using the OBE and BOPPPS teaching models (87.9 ± 7.9) were significantly higher than those of students enrolled in the 2017 class using the traditional teaching model (78.7 ± 14) (*P* < 0.05, Fig. 4).

**Fig. 5:**
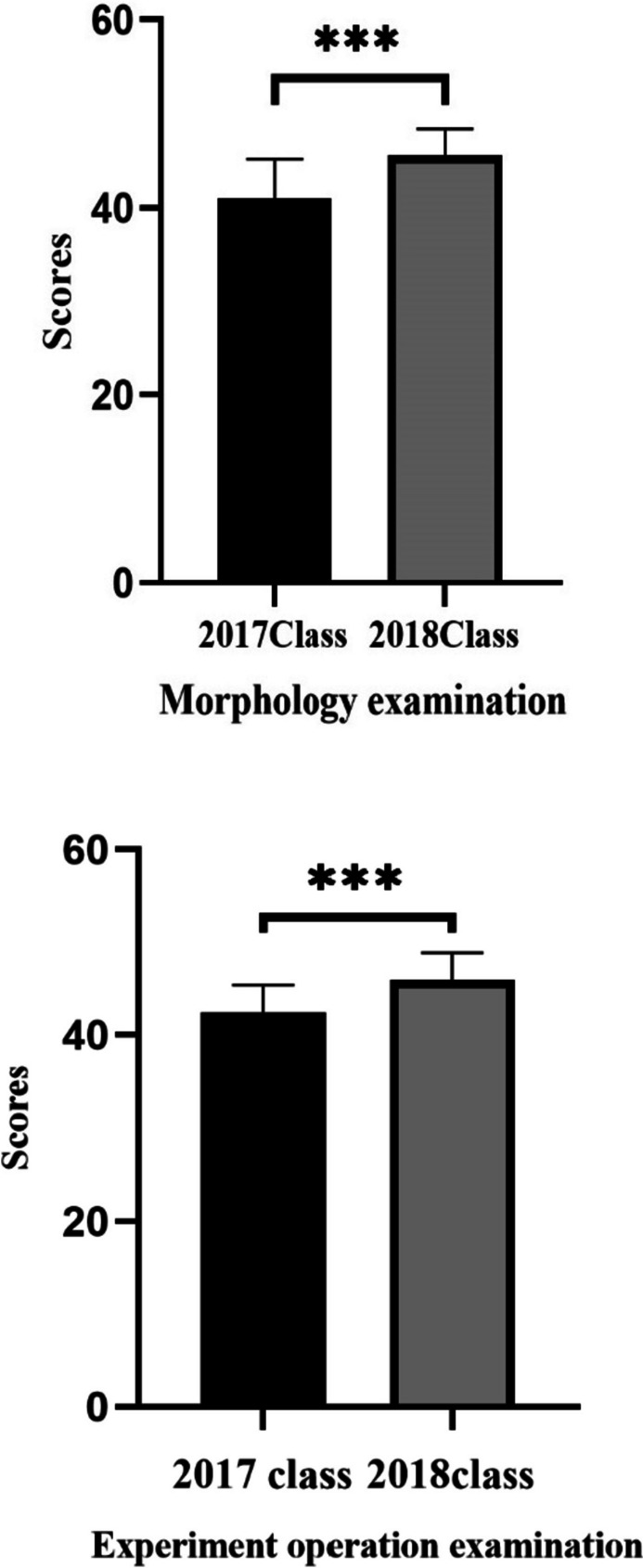
The difference of experiment operation examination and morphology examination. ** *P* < 0.05 2018 class compared to 2017 class

The experimental examination results of the experimental group and the control group were compared and analyzed, and the experimental operation examination (50 points) and morphology examination (50 points) were used as the judgment criteria. The experimental operation examination and morphology examination scores of the 2018 class were higher than those of the 2017 class, and this difference was statistically significant (*P* < 0.05, Fig. 5).

**Fig. 6:**
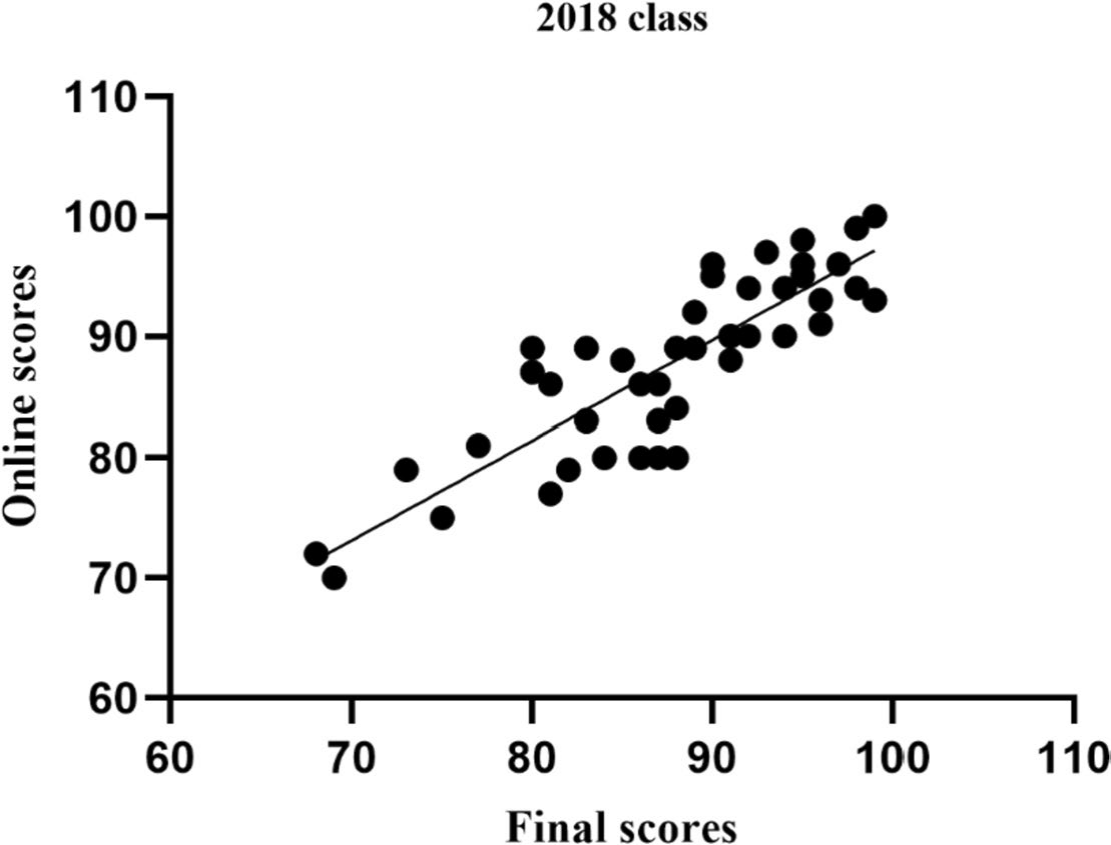
Correlation between fnal score and the online score

The correlation between online score and final total score was also analyzed; the online score was positively correlated with the total test score (R2 = 0.74). Students' independent learning ability was therefore improved. This assessment method thus promotes students' digestion and absorption of theoretical knowledge to improve their academic performance (*P* < 0.05, Fig. 6).

### The degree of objective attainment

The students in the 2017 class did not conduct a large degree of analysis and evaluation of curriculum objectives and remained at the level of fuzzy qualitative analysis, so they could not make a clear judgment. The students in the 2018 class, however, implemented OBE-BOPPPS teaching, and in the initial design, the weight of the curriculum evaluation composition corresponded to the training objectives one by one. Finally, the three course objectives of this course were analyzed as follows:

- Objective 1: To master the preparation of blood smears and Wright's staining method, Wright's staining principle and quality assurance.
- Objective 2: Blood smear preparation and staining and peripheral blood cell morphology.
- Objective 3: To cultivate students' rigor and responsibility in their work and drive them to constantly explore the spirit of innovation.

**Fig. 7:**
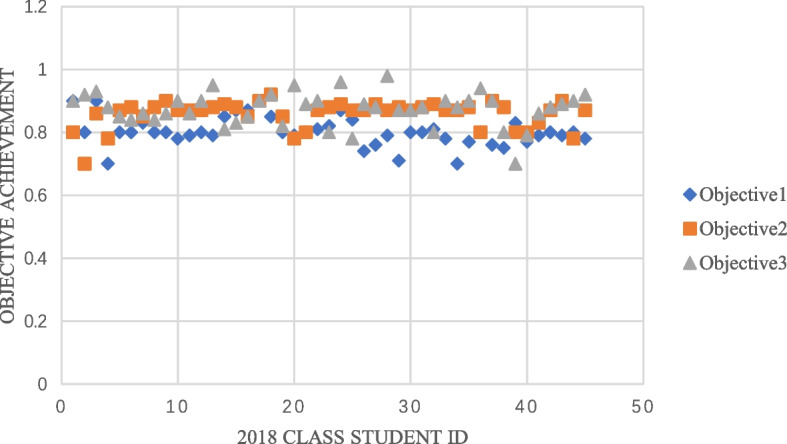
Achievement of curriculum objectives

Overall, the average attainment of objective 1 was 0.8, that of objective 2 was 0.86, and that of objective 3 was 0.87 (Fig. 7). In the knowledge target part, the students' understanding of abstract principles and concepts remained unclear, and their understanding of rote memorization was not thorough enough. Teachers need to focus on these students and help them. There was no significant difference between the attainment of objective 2 and objective 3. The students were easily able to carry out the experimental operation, carefully and seriously observe the morphology, exhibit divergent clinical thinking, and create some comprehensive experiments combined with clinical practices.

### Student questionnaire survey

To explore the students' acceptance of the teaching model, a questionnaire survey of the course experience was issued after their class (Table 2). A total of 45 copies were distributed to all students in the 2018 class, yielding 100% recovery and effective rates. The results of the questionnaire survey show that 95% of the students approve of this teaching model and that students like to participate in classroom teaching and teacher–student interaction. In this class, 90% of the students believed their interest in learning and their self-learning ability improved, their technical principles and morphological understanding of the knowledge deepened, and their ability to find problems, then solve and analyze them and their clinical logical thinking ability and adaptability all also improved. More than 85% of the students thought that their active learning ability had been strengthened and that their learning efficiency had been improved.

**Fig. 8:**
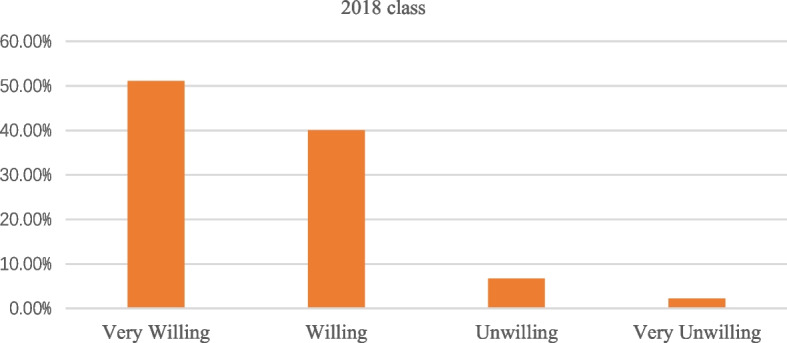
Survey result on whether students are willing to continue to implement BOPPPS teaching model

Regarding the teaching and research work of the Myeloid cytology test in the next semester, this paper surveyed whether students preferred the BOPPPS teaching model or the traditional teaching model and whether students were willing to adopt the BOPPPS model in the practical teaching of the Myeloid cytology course in their next semester (Fig. 8). More than 90% of the students were willing to accept the BOPPPS teaching model in the experimental teaching of myeloid cytology tests in the next semester.

## Discussion

### Curriculum evaluation

Clinical Basic Laboratory Technology is a very practical course that requires a combination of theory and practice. Therefore, its curriculum evaluation requires diversified reform. The traditional examination is a written test, but a test cannot fully reflect the learning effect on students. This study was conducted through a combination of preclass, in-class and postclass data. Through the assessment and evaluation method, weight was given to the score on each assessment index to integrate the hundred-mark system as the final academic performance. This diversified assessment and evaluation made the final examination scores and experimental assessment of the 2018 class significantly better than those of the 2017 class with traditional examination methods. Through the results of the diversified assessment, deficiencies in students' basic knowledge and professional knowledge were found, and the corresponding problems were rectified. Only by discovering problems, from "teaching" to "learning", can teaching be improved.

Hence, the degree of goal attainment shows that whether the goal of this teaching content meets the expected requirements of teachers or not, individualized guidance and evaluation should be given to students according to their problems. At the same time, it is necessary to consider whether it is reasonable to set up a questionnaire and to consider the real feedback of students, which is conducive to communication and mutual trust between teachers and students. Teachers adjust their teaching plan according to the degree of students' attainment of goals. In view of the weak links between students' abstract principles and concepts, how to make students more interested and engaged in understanding entails that teachers need to rethink and solve these problems, change their teaching methods and teaching content design, and better grasp their learning content according to the learning characteristics of each student.

### OBE + BOPPPS blended teaching

To change the traditional classroom teaching model, with the popularization of digital networks and the development of information technology, the learning initiative for the students themselves should make full use of the information platform.

Students can make full use of the information platform by focusing on the OBE concept and adopting the BOPPPS teaching method. The above research shows that the effect of teaching experiments is stronger than that of traditional experiment teaching. Teachers complete online learning, classroom discussion interaction, after-school homework, and knowledge expansion while maintaining students' autonomy, forming an evaluation-feedback-improvement closed-loop model.

The BOPPPS mode increases the complete teaching resources for online mobile learning. Teachers use online platform resources to overcome the time and space constraints of offline teaching so that students can develop good self-study habits. Without a teacher on the scene, students can also feel clear learning objectives, an immersive learning atmosphere, learning enthusiasm for curriculum knowledge, and experience independent learning [16]. They participate in the whole course teaching session in the form of discussion, question and answer, questionnaire, and so on. Such interactivity improves students' enthusiasm, promoting their active learning participation while improving the classroom teaching atmosphere. The whole teaching process highlights the learning objectives of teaching and classroom participation and promotes the organic unification of teaching–learning objectives and professional training objectives. On the one hand, through whole course and multidimensional assessment and evaluation, teachers are encouraged to improve their practice teaching throughout all teaching activities, which can urge them to improve their teaching strategies and enhance their teaching abilities. On the other hand, it can stimulate students' learning enthusiasm and participation, enhance their sense of learning acquisition, and, finally, improve the quality of teaching practice.

The questionnaire survey results show that the classroom highlights students' real participatory learning and enables them to explore and think independently, experience the fun of learning, and improve their understanding of morphology and autonomous learning ability. The training of medical laboratory students' clinical thinking ability of independent thinking, determining problems, and solving problems enhances their innovation consciousness and operation ability. Moreover, their language expression ability and clinical communication ability are improved, laying the foundation for subsequent practice and work.

### Teacher requirements

The BOPPPS teaching model based on the OBE concept entails higher requirements for teachers' professional teaching levels. Teachers are no longer installers of knowledge but guides of knowledge, process supervisors, and evaluation participants. The BOPPPS teaching model also has higher requirements for teaching quality. Teachers should not only have a solid theoretical foundation but also have rich practical experience. At the same time, it is necessary for them to communicate with students in time according to the problems they encounter in learning and adjust their teaching content as students master their learning situation to improve the teaching level of both teachers and the clinical teaching level and better mobilize the enthusiasm of students. Teachers, in the requirements of the syllabus, the teaching objectives, and the preclass preview stage of teaching design need to add vivid, interesting microvideos. As a result, the teaching model requires teachers to spend a certain amount of time and energy to participate in the preparation of their lessons; the clinical basic laboratory technology course is undertaken by the senior title teachers of the hospital. I not only need to complete the daily hospital work but also go to the university to complete the professional course teaching tasks. Therefore, how can OBE be better integrated with BOPPPS? How can teachers balance their daily clinical work and the curriculum? Finally, how can the overload of teachers with the task due to overreliance on lecture-based teaching be prevented? Completing the task of teaching design needs further research and discussion in the future.

## Conclusion

The BOPPPS model based on the OBE concept initially showed effective teaching results in the research and practice of basic clinical laboratory practice teaching. It aims to improve students' interest in learning and ability, cultivate their innovative spirit, improve their practical ability, satisfy students' learning needs to achieve the 'student-centered' teaching purpose. There are also deficiencies that need to be further improved in the teaching process, e.g., networking equipment still needs to be improved, curriculum teaching needs more scientific and reasonable designs, to ensure optimization and continuous improvement.

## Data Availability

All data generated or analysed during this study are included in this manuscript.

## Abbreviations

BOPPPS: A teaching method composed of six elements including bridge-in, learning objective, pretest, participatory learning, posttest and summary
OBE: Outcome-based education

## References

[1] Zeng CX, Meng CX, Li YX (2022). Innovating teaching concepts to improve the training quality of medical laboratory talents [J]. Contin Med Educ China.

[2] Ding HM, Zang XL, Zhao ZJ (2019). Discussion on the improvement of clinical blood morphology teaching in medical laboratory specialty [J]. Int J Lab Med.

[3] Li P, Liu Y, Liu SQ (2022). Research on basic inspection teaching of OBE combined with blended teaching [J]. Contin Med Educ China.

[4] Sasipraba T, KajaBanthaNavas R, Nandhitha NM (2020). Assessment tools and rubrics for evaluating the capstone projects in outcome based education [J]. Procedia Comput Sci.

[5] Huang CL, Sheng GJ, Lu YX et al. Construction of information talent training mode system based on OBE [J]. China Education Informationization. 2019(23):31–34.

[6] Zhang NX (2019). Promoting the quality of professional education in colleges and universities with OBE concept [J]. Univ Educ.

[7] Bai LY, Fan SJ, Zhao J (2019). Reform of physical geodesy course based on OBE [J]. Adv J.

[8] Zheng DX, Huang JH (2018). Exploration on the reform of OBE-oriented classroom teaching model in application-oriented universities [J]. Softw Eng.

[9] Jajie Y, Jie Y, Junrong WM (2019). The effect of microteaching combined with the BOPPPS model on dental materials education for predoctoral dental students[J]. J Dent Educ.

[10] Ling MY, Hu Y, Jin WX (2022). Teaching design of ' biochemistry experiment ' based on BOPPPS model [J]. Agric Prod Process.

[11] Li J, Chen HZ, Xu GC (2022). Application of BOPPPS teaching model in simulation teaching of western diagnostics [J]. J Clin Chin Med.

[12] Zheng XD, Luo XA, Lai YZ (2022). Application and exploration of OBE + BOPPPS teaching model in orthodontics teaching under the background of " new medicine " [J]. Chin Med Educ Technol.

[13] Shen XY (2022). Application of OBE-BOPPS teaching model in pathogenic biology and immunology teaching [J]. Health-related Vocational Education.

[14] Hu K, Ma RJ, Ma C (2022). Comparison of the BOPPPS model and traditional instructional approaches in the thoracis surgery education[J]. BMC Med Educ.

[15] Liu J, Zhou MY (2022). Research and practice of BOPPPS teaching model of medical information literacy course from the perspective of OBE [J]. Chin J Tradit Chin Med Libr Inform.

[16] Jiao YY, Pan XG, Ma MH (2020). Improved BOPPPS model teaching design in undergraduate online teaching [J]. J High Educ Res.

